# Solution Combustion Synthesis and Characterization of (CoCrFeMnNi)_3_O_4_ High-Entropy Oxide Using Different Fuels

**DOI:** 10.1021/acsomega.5c12933

**Published:** 2026-03-28

**Authors:** Umay Cinarli Yavas, Ahmet Turan

**Affiliations:** Materials Science and Nanotechnology Engineering Department, Faculty of Engineering, 683672 Yeditepe University, Ataşehir, Istanbul 34755, Turkey

## Abstract

High-entropy oxides (HEOs) have recently attracted significant interest due to their tunable crystal structures, compositional versatility, and promising functional properties in energy storage, catalysis, and magnetic applications. Among various synthesis routes, solution combustion synthesis (SCS) offers a rapid and energy-efficient pathway for producing phase-pure HEO powders with controlled morphology. In this study, (CoCrFeMnNi)_3_O_4_ high-entropy oxide was synthesized via SCS using three different fuels, followed by postcombustion heat treatments at 800, 900, and 1000 °C for 1 h. The calcination temperatures were selected based on thermogravimetric analysis (TGA) and literature data. Phase formation, microstructural evolution, and elemental distribution were investigated by X-ray diffraction (XRD) and scanning electron microscopy coupled with energy-dispersive spectroscopy (SEM-EDS), while specific surface area and porosity characteristics of selected samples were evaluated by Brunauer–Emmett–Teller (BET) analysis and its corresponding Barrett–Joyner–Halenda (BJH) method. The results revealed that the choice of fuel significantly influenced the combustion characteristics, phase purity, and particle morphology, while the calcination temperature played a key role in grain growth and densification. Glycine at a stoichiometric ratio (Φ_e_ = 1) and calcination at 900 °C yielded the most favorable results, producing sharp spinel peaks consistent with the $Fd\overset{-}{3}m$ space group and elemental distributions closest to equimolar. TGA confirmed high thermal stability with <3% weight loss up to 1000 °C, while the citric acid route exhibited ∼25% mass loss due to residual organics. Postcalcination SEM analyses showed homogeneous microstructures with well-defined grains, particularly in glycine-derived samples, whereas excess fuel or unsuitable stoichiometry led to porous or amorphous products. BET/BJH analyses of glycine-derived samples prepared at the 1.0× fuel stoichiometry further confirmed the temperature-dependent textural evolution, showing a progressive reduction in specific surface area and porosity with increasing calcination temperature, in agreement with SEM-observed densification. This work provides a systematic comparison of fuel-dependent SCS synthesis for (CoCrFeMnNi)_3_O_4_ and establishes a synthesis parameter space for obtaining single-phase spinel oxides with controlled microstructures at relatively low processing temperatures.

## Graphical abstract

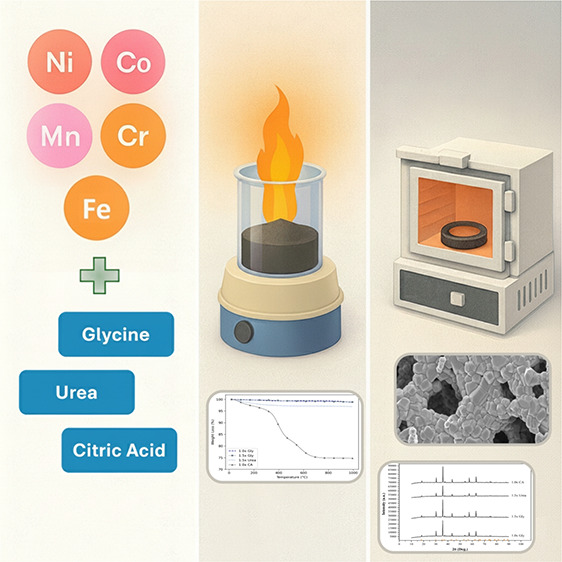

## 1 Introduction

The continuous evolution of materials design has been driven by the need to achieve advanced combinations of mechanical, thermal, and chemical properties. Traditional alloy and ceramic systems, which are typically based on one principal component with minor alloying additions, often face intrinsic limitations in achieving multifunctionality. In recent decades, this limitation has been addressed through the emergence of high-entropy alloys (HEAs), a class of materials composed of five or more principal elements in equimolar or near-equimolar ratios. Although multicomponent alloys were explored historically, the systematic development of HEAs began in the early 2000s with the pioneering works of Yeh et al. and Cantor et al., who demonstrated that high configurational entropy could stabilize simple solid-solution phases instead of complex intermetallic. HEAs are generally defined by compositions in which each element occupies 5–35 at. %, thereby expanding the compositional design space and enabling the tailoring of microstructure and properties through elemental synergy.^1–4^

The thermodynamic basis underlying this multicomponent design strategy is the concept of mixing entropy (Δ*S*_mix_), which plays a central role in governing phase formation and stability in such systems. Δ*S*_mix_ is one of the fundamental thermodynamic parameters governing the formation and stability of high-entropy materials. For an n-component system, Δ*S*_mix_ is defined in eq 1 as^5^

$$\Delta_{S_{\mathrm{mix}}}=-R\sum_{i=1}^{n}c_{i}\;\ln\;c_{i}=R\;\ln\;n$$ 1

where *R* denotes the gas constant and *c*_*i*_ is the molar fraction of the *i*-th element. In an equimolar composition (*c*_*i*_ = 1/*n*), this expression simplifies to Δ*S*_mix_ = *R* ln­(*n*), indicating that Δ*S*_mix_ increases logarithmically with the number of constituent species. This relation provides a quantitative basis for classifying multicomponent systems according to their configurational entropy. In particular, when Δ*S*_mix_ exceeds 1.5*R*, the system is considered to possess sufficiently high configurational entropy to thermodynamically favor a random solid-solution state over ordered or multiphase configurations.^6^ This entropy-driven stabilization, together with lattice distortion, sluggish diffusion, and the so-called cocktail effect, gives rise to the exceptional mechanical, thermal, electrical, and magnetic properties reported for HEAs.^2^

The high-entropy design concept has subsequently been extended from metallic systems to ceramics, leading to the development of high-entropy oxides (HEOs). HEOs are typically single-phase oxide solid solutions in which multiple cations occupy equivalent crystallographic sites in near-equimolar proportions. The first experimental demonstration was reported by Rost et al. in 2015 for the rock-salt structured (MgCoNiCuZn)­O, which exhibited remarkable phase stability despite its chemical complexity. This discovery initiated extensive research into HEOs with diverse crystal structures, including rock-salt, fluorite, perovskite, and spinel. Subsequent studies revealed that HEOs can exhibit ultralow thermal conductivity, tunable magnetic behavior, high dielectric constants, and promising electrochemical performance, properties that are closely linked to cation disorder and entropy stabilization.^7–9^

Among the various crystal structures, spinel-type high-entropy oxides with the general formula AB_2_O_4_ have attracted particular attention due to their structural flexibility and multifunctional potential. The spinel structure accommodates cations with different valence states and ionic radii, enabling fine control over electronic, magnetic, and electrochemical properties. As a result, spinel HEOs have been investigated for applications in lithium-ion batteries, supercapacitors, catalysis, and magnetic devices.^10,11^ In particular, (CoCrFeMnNi)_3_O_4_ has emerged as a model high-entropy spinel system, exhibiting structural stability, magnetic ordering, and electrochemical activity.

High-entropy oxides can be synthesized via a variety of routes, including solid-state reactions, mechanical alloying, sol–gel processing, hydrothermal synthesis, and vapor-phase techniques. While solid-state methods are scalable and widely used, they typically require high temperatures and long dwell times to achieve compositional homogeneity. In contrast, SCS offers a rapid, energy-efficient alternative that enables molecular-level mixing of cations and the formation of nanostructured oxides in a single processing step.^12–14^ In SCS, an exothermic redox reaction between metal nitrates (oxidizers) and an organic fuel generates sufficient heat to drive oxide formation, often within seconds. The combustion behavior, including flame temperature, gas evolution, and reaction kinetics, is strongly governed by fuel chemistry and fuel-to-oxidizer ratio.

Combustion synthesis can be classified into self-propagating high-temperature synthesis (SHS), volume combustion synthesis (VCS), and SCS. Among these, SCS is particularly suitable for synthesizing complex oxides because the aqueous precursor solution allows homogeneous cation distribution prior to ignition. The combustion reaction proceeds only when the heat released exceeds the ignition requirement, and depending on reaction conditions, it may occur in flaming, smoldering, or explosive modes. These combustion regimes directly influence crystallinity, particle size, porosity, and defect chemistry.^15^

A practical way to parametrize mixtures is via the equivalence ratio Φ_e_, defined from elemental stoichiometry (eq 2); Φ_e_ = 1 denotes a stoichiometric mixture, Φ_e_ > 1 fuel-lean, and Φ_e_ < 1 fuel-rich. For Φ_e_ = 1, the required oxidizer/fuel molar ratio (O/F) is obtained by dividing the total oxidizing and reducing valences of the oxidizer by those of the fuel. In this calculation, oxygen is the only oxidizing element; Carbon, hydrogen and metal cations are reducing elements. Nitrogen is neutral. Oxidizing elements have a positive valence value, while reducing elements have a negative valence value.^12^

$$\Phi_{e}=\frac{\sum (\mathrm{Number}\;\mathrm{of}\;\mathrm{oxidizing}\;\mathrm{elemen}ts)\times (\mathrm{Valence}\;\mathrm{value})}{-1\sum (\mathrm{Number}\;\mathrm{of}\;\mathrm{reducing}\;\mathrm{elements})\times (\mathrm{Valence}\;\mathrm{value})}$$ 2

There are several uses for the fuels utilized in the SCS process. Carbon dioxide (CO_2_) and water (H_2_O) gas molecules are produced during combustion, releasing heat. These fuels are typically sources of carbon (C) and hydrogen (H). Additionally, by forming intricate structures with the metal ions in solution, the fuels enable the cations to mix uniformly. These fuels can also break down into their constituent parts and dissolve in a solution, releasing combustible gases such NH_3_ and HNCO that react with NO_*x*_. Certain characteristics are necessary for a fuel (such as urea and glycine) to be acceptable for SCS. Among these characteristics are the presence of N–N bonding compounds, water solubility, a low ignition temperature (<500 °C), compatibility with metal nitrates (i.e., structure that prevents an explosion in a controlled combustion reaction), and a low molecular weight during combustion. It is simple to locate or prepare, and it releases innocuous gases. These characteristics are critical to the SCS method’s successful implementation.^12^

The versatility of the high-entropy concept in oxide systems has been demonstrated by numerous studies focusing on both structural diversity and functional performance. Dabrowa et al. reported single-phase spinel (CoCrFeMnNi)_3_O_4_ and compared it with rock-salt-type (CoCuMgNiZn)­O, confirming compositional homogeneity and reporting lattice parameters of 8.355Å and 4.236Å, respectively.^16^ Sarkar et al. demonstrated that (Co_0.2_Cu_0.2_Mg_0.2_Ni_0.2_Zn_0.2_)­O anodes exhibited stable cycling and reversible capacity over 500 charge–discharge cycles due to entropy-driven phase stabilization.^17^ Low-temperature synthesis routes have also been explored for spinel high-entropy oxides. Simsek et al. synthesized spinel (FeNiCoCuZn)_3_O_4_ below 100 °C, obtaining nanocrystalline $Fd\overset{-}{3}m$ structures with stable lithium storage, oxygen evolution activity, paramagnetic behavior, and a band gap of 2.2–2.5 eV.^18^ Mao and co-workers employed solution combustion synthesis to produce nanocrystalline (CoCrFeMnNi)_3_O_4_ spinel and reported ferrimagnetic behavior at room temperature, with magnetic properties tunable through partial substitution of nonmagnetic Zn^2+^ for Co^2+^ or Ni^2+^.^19^ Beyond magnetic properties, multicomponent spinel oxides have been investigated for electrochemical energy storage. Chen et al. achieved high-rate cycling stability in (MgTiZnCuFe)_3_O_4_ anodes, while Talluri et al. developed (CrMnFeCoNi)_3_O_4_-based supercapacitor electrodes with high capacitance and long-term stability.^20,21^ Petrovičovà et al. further improved electrochemical performance through electrospinning and sol–gel synthesis combined with lithium doping and carbon incorporation.^22^ More recently, Wang et al. highlighted the role of multication synergy, known as the cocktail effect, in enhancing electrochemical stability and dendritic nanostructure formation in Mg–Co–Ni–Cu-Zn high-entropy oxides.^23^ A comparative overview of representative high-entropy oxide compositions reported in the literature is summarized in Table 1.

**Table 1:** Comparative Summary of High-Entropy Oxide Studies in the Literature

author(s), year	composition	crystal structure	synthesis method	reference
Dabrowa et al., 2018	(CoCrFeMnNi)_3_O_4_	spinel	solid-state	^16^
Mao et al., 2019	(CoCrFeMnNi)_3_O_4_	spinel	solution combustion synthesis (SCS)	^19^
Musicó et al., 2019	(Cr_0.2_Mn_0.2_Fe_0.2_Co_0.2_Ni_0.2_)_3_O_4_	spinel	solid-state	^24^
Wang et al., 2020	(FeCoNiCrMn)_3_O_4_	spinel	solid-state	^25^
Grzesi̇k et al., 2020	(Co,Cr,Fe,Mn,Ni)_3_O_4_	spinel	solid-state	^26^
Talluri et al., 2021	(CrMnFeCoNi)_3_O_4_	spinel	reverse coprecipitation	^21^
Petrovičovà et al., 2022	(Cr_0.2_Mn_0.2_Fe_0.2_Co_0.2_Ni_0.2_)_3_O_4_	spinel	electrospinning/sol–gel	^22^
Li et al., 2023	(Co_0.2_Cr_0.2_Fe_0.2_Mn_0.2_Ni_0.2_)_3_O_4_	spinel	polyacrylamide gel method	^27^
Sun et al., 2022	(Co,Cr,Fe,Mn,Ni)_3_O_4_	spinel	surfactant-assisted hydrothermal technology combined with a heat treatment method	^28^
He et al., 2023	(CoCrFeMnNi)_3_O_4_	spinel	solid-state	^29^
Xie et al., 2025	Pt/(CoMnFeCrNi)_3_O_4_	spinel	solution combustion synthesis (SCS) + atomic layer deposition (Pt)	^30^

In this study, high-entropy spinel oxide (CoCrFeMnNi)_3_O_4_ was synthesized in this study using SCS. The resulting material crystallizes in the AB_2_O_4_ spinel structure with the $Fd\overset{-}{3}m$ space group, and glycine, urea, and citric acid were employed as fuels with systematically varied stoichiometries. The combustion behavior, reaction kinetics, phase formation, microstructural evolution, and elemental distribution were systematically investigated to elucidate the role of fuel chemistry and fuel ratio in governing phase stability and crystallization. The as-synthesized powders were subsequently calcined at 800, 900, and 1000 for 1 h, with the calcination temperatures selected based on thermogravimetric analysis (25–1000 °C, N_2_ atmosphere) and relevant literature. Through this systematic approach, the present study aims to define the synthesis window and limitations of SCS for obtaining single-phase (CoCrFeMnNi)_3_O_4_ spinels at relatively low processing temperatures, while providing a robust experimental framework for future application-oriented investigations.

## 2 Materials and Methods

In the present study, high-entropy spinel oxide (CoCrFeMnNi)_3_O_4_ was synthesized by the SCS method using different fuel types and fuel stoichiometries (Figure 1). Glycine, urea, and citric acid were used as fuels, and their effects on phase formation and microstructural evolution were systematically investigated. SCS experiments were conducted at 250 °C in both a magnetic stirrer and a heating mantle, followed by postcalcination treatments at 800, 900, and 1000 °C for 1 h to enhance crystallinity and phase stability.

**Figure 1:**
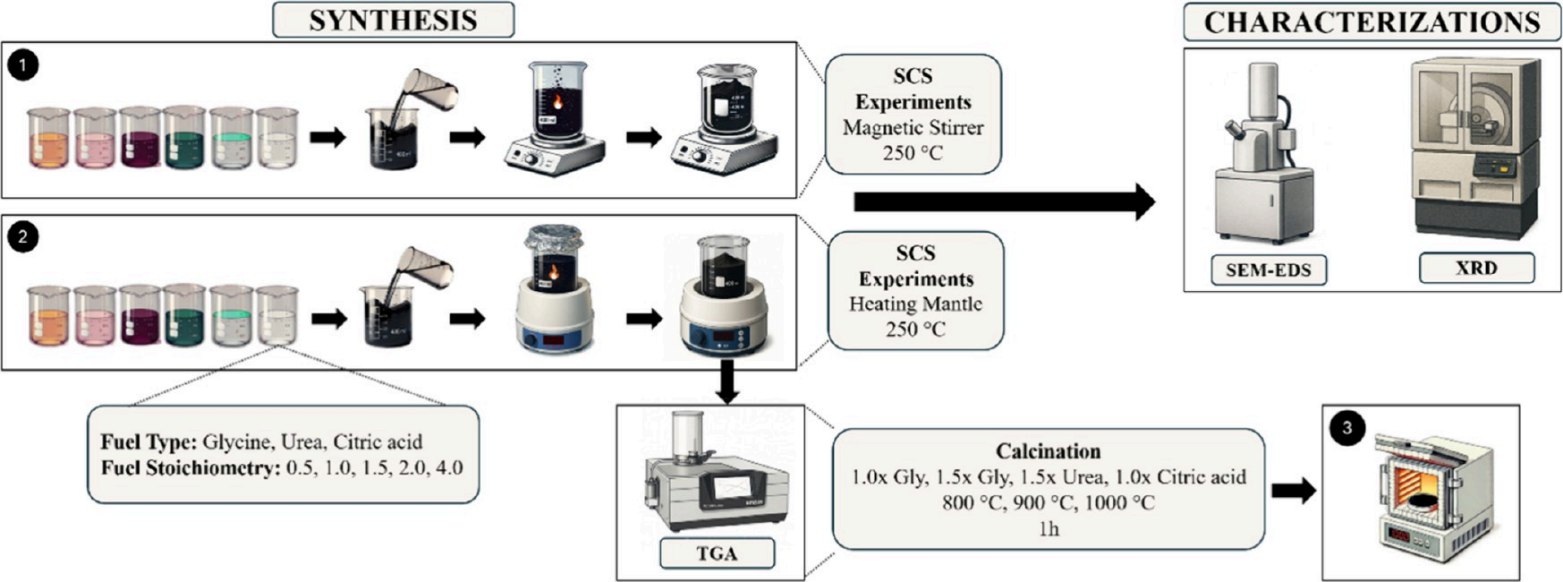
Schematic representation of the experimental procedure.

Φ_e_ number calculation is conducted using a specific approach based on the calculation of the reducing and oxidizing valences of the redox mixture (eq 2). In this method, carbon, hydrogen and metals are treated as reducing elements, with their respective valences defined as carbon (+4), hydrogen (+1) and metal (*v*). Oxygen acts as the oxidizing element with a valence of (−2), while nitrogen is assumed to have a valence of zero. For instance, the reducing valence of glycine (C_2_H_5_NO_2_) is calculated as [(4 × 2)+(5 × 1)+(1 × 0)+(2 × (−2))] = +9. Similarly, the oxidizing valence of a metal nitrate, Me_*v*_(NO_3_)_*v*_, is determined as [*v* + (3*v* × (−2))] = −5v.^31–33^ In the SCS experiments carried out with glycine, urea and citric acid at fuel ratios of 0.5×, 1.0×, 1.5×, 2.0× and 4.0×, the Φ_e_ values were determined as 2, 1, 0.6, 0.5 and 0.25, respectively, using eq 2.

### 2.1 SCS Experiments with the Heated Magnetic Stirrer

All nitrate raw materials used in SCS experiments were of Merck quality. Isolab extra pure glycine (≥98.5%, C_2_H_5_NO_2_), Sigma urea (≥98.0%, CH_4_N_2_O) and Isolab citric acid (≥99.7%, C_6_H_8_O_7_) were used as fuel. All solution combustion syntheses were carried out on an Onilab MS-H280 Pro model heated magnetic stirrer.

The reaction equations were given below (eq 3–5) were used to obtain (CoCrFeMnNi)_3_O_4_ through SCS with glycine, urea and citric acid, respectively.

$$9\mathrm{Co}{\left({\mathrm{NO}}_{3}\right)}_{2}.6H_{2}O+9\mathrm{Cr}{\left({\mathrm{NO}}_{3}\right)}_{3}.9H_{2}O+9\mathrm{Fe}{\left({\mathrm{NO}}_{3}\right)}_{3}.9H_{2}O+9\mathrm{Mn}{\left({\mathrm{NO}}_{3}\right)}_{2}.4H_{2}O+9\mathrm{Ni}{\left({\mathrm{NO}}_{3}\right)}_{2}.6H_{2}O+60C_{2}H_{5}{\mathrm{NO}}_{2}=3{\left(\mathrm{CoCrFeMnNi}\right)}_{3}O_{4}+\mathrm{Water}+\mathrm{Gases}$$ 3

$$6\mathrm{Co}{\left({\mathrm{NO}}_{3}\right)}_{2}.6H_{2}O+6\mathrm{Cr}{\left({\mathrm{NO}}_{3}\right)}_{3}.9H_{2}O+6\mathrm{Fe}{\left({\mathrm{NO}}_{3}\right)}_{3}.9H_{2}O+6\mathrm{Mn}{\left({\mathrm{NO}}_{3}\right)}_{2}.4H_{2}O+6\mathrm{Ni}{\left({\mathrm{NO}}_{3}\right)}_{2}.6H_{2}O+60{\mathrm{CH}}_{4}N_{2}O=2{\left(\mathrm{CoCrFeMnNi}\right)}_{3}O_{4}+\mathrm{Water}+\mathrm{Gases}$$ 4

$$18\mathrm{Co}{\left({\mathrm{NO}}_{3}\right)}_{2}.6H_{2}O+18\mathrm{Cr}{\left({\mathrm{NO}}_{3}\right)}_{3}.9H_{2}O+18\mathrm{Fe}{\left({\mathrm{NO}}_{3}\right)}_{3}.9H_{2}O+18\mathrm{Mn}{\left({\mathrm{NO}}_{3}\right)}_{2}.4H_{2}O+18\mathrm{Ni}{\left({\mathrm{NO}}_{3}\right)}_{2}.6H_{2}O+60C_{6}H_{8}O_{7}=6{\left(\mathrm{CoCrFeMnNi}\right)}_{3}O_{4}+\mathrm{Water}+\mathrm{Gases}$$ 5

Assuming that the amount of (CoCrFeMnNi)_3_O_4_ obtained as a result of the SCS reaction was 1 g, the raw materials reacted were calculated. Experiments were conducted using fuel stoichiometries of 0.5×, 1.0×, 1.5×, 2.0×, and 4.0× for glycine, urea, and citric acid, while keeping the total molar amount of metal nitrates constant at 0.00330 mol for each metal (Table 2). Under these constant oxidizer conditions, systematic variations in fuel stoichiometry modify the combustion temperature, gas evolution rate, and reaction completeness, thereby directly influencing the final product yield.

**Table 2:** Amounts of Oxidizers and Fuels Used in Solution Combustion Synthesis at Different Fuel Stoichiometries

	fuel		fuel	Co(NO_3_)_2_ .6H_2_O	Cr(NO_3_)_3_.9H_2_O	Fe(NO_3_)_3_.9H_2_O	Mn(NO_3_)_2_ .4H_2_O	Ni(NO_3_)_2_.6H_2_O
	stoichiometry	Φ_e_	amount					
type	(x)		(g)	(g)	(g)	(g)	(g)	(g)
glycine	0.5	2.00	0.829	0.960	1.320	1.333	0.828	0.959
glycine	1.0	1.00	1.658	0.960	1.320	1.333	0.828	0.959
glycine	1.5	0.67	2.486	0.960	1.320	1.333	0.828	0.959
glycine	2.0	0.50	3.315	0.960	1.320	1.333	0.828	0.959
glycine	4.0	0.25	6.63	0.960	1.320	1.333	0.828	0.959
urea	0.5	2.00	0.993	0.960	1.320	1.333	0.828	0.959
urea	1.0	1.00	1.986	0.960	1.320	1.333	0.828	0.959
urea	1.5	0.67	2.979	0.960	1.320	1.333	0.828	0.959
urea	2.0	0.50	3.972	0.960	1.320	1.333	0.828	0.959
urea	4.0	0.25	7.944	0.960	1.320	1.333	0.828	0.959
citric acid	0.5	2.00	1.056	0.960	1.320	1.333	0.828	0.959
citric acid	1.0	1.00	2.112	0.960	1.320	1.333	0.828	0.959
citric acid	1.5	0.67	3.168	0.960	1.320	1.333	0.828	0.959
citric acid	2.0	0.50	4.224	0.960	1.320	1.333	0.828	0.959
citric acid	4.0	0.25	8.448	0.960	1.320	1.333	0.828	0.959

The temperature was set at 250 °C and, distilled water was used to prepare the aqueous solutions. Solid oxidizing raw materials were dissolved in 10 mL of distilled water and fuel was dissolved in approximately 5 mL of distilled water (Figure 2). After the oxidizing raw materials and glycine were dissolved in separate beakers with distilled water, they were transferred to another beaker and mixed. The resulting aqueous solution was placed on a heated magnetic stirrer set at 250 °C. After about 20 min, most of the water was removed and the combustion reaction occurred.

**Figure 2:**
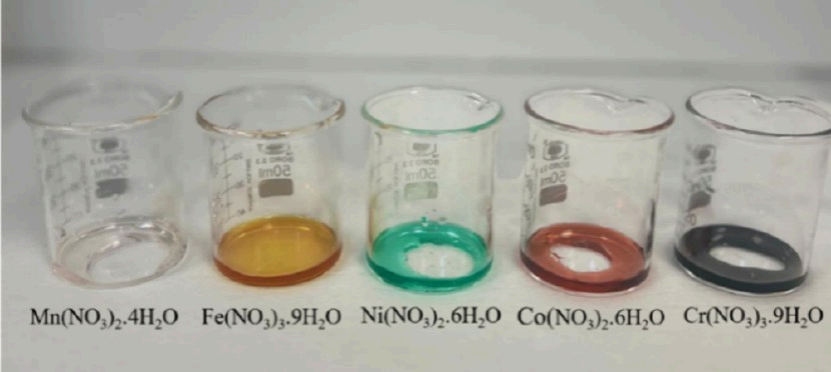
Aqueous solutions prepared for the SCS experiments.

Experiments with different fuel types and ratios allowed systematic investigation of the roles of glycine, urea and citric acid as fuels in the combustion synthesis process. Thus, the effects of each fuel type on the reaction results were analyzed in detail and a comparative evaluation was made possible.

### 2.2 SCS Experiments with the Heating Mantle

In the SCS experiments carried out with different glycine, urea and citric acid ratios in the heated magnetic stirrer, the combustion reactions were repeated in the heating mantle to ensure homogeneous heat distribution (Figure 3). Heating mantle experiments were conducted using stoichiometric fuel ratios of 0.5×, 1.0×, 1.5×, 2.0×, and 4.0× for glycine, 1.0× and 1.5× for urea, and 0.5×, 1.0×, and 1.5× for citric acid, enabling a systematic evaluation of each fuel’s performance under controlled conditions.

**Figure 3:**
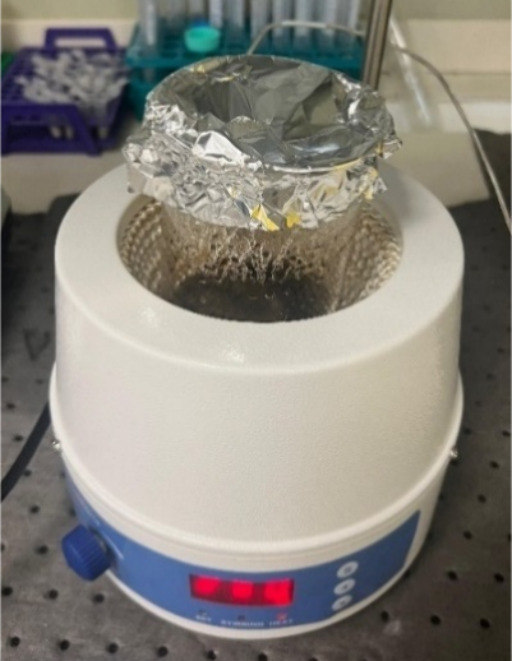
SCS experiment performed on the heating mantle.

All nitrate raw materials used in the SCS experiments were of Merck-grade quality. High-purity fuels, as same as the experimental series on the heated magnetic stirrer, were employed in the SCS experiments. To maintain optimal conditions and precision, all solution combustion synthesis experiments were conducted using an a Thermomac brand HMS500D model heating mantle.

### 2.3 Calcination of Products Obtained through SCS

The products obtained by SCS evaluated using XRD, SEM-EDS reaction parameters, and samples with optimum properties were selected (Table 3). TGA was performed on a Netzsch TG 309 Libra model under nitrogen atmosphere between 25 and 1000 °C to determine calcination temperatures. Calcination samples were selected according to TGA results were calcined in a Protherm brand PLF 160/5 model muffle furnace at 800, 900 and 1000 °C for 1 h each.

**Table 3:** Calcination Parameters of the SCS Samples

fuel type	fuel stoichiometry	calcination temperature	duration (h)
glycine	1.0×	800 °C, 900 °C, 1000 °C	1
	1.5×		
urea	1.5×		
citric acid	1.0×		

The products obtained after SCS experiments with different fuel types and ratios and after calcination were characterized by SEM, EDS, XRD and BET/BJH analysis. SEM and EDS analyses were performed using Jeol brand JCM7000 model and ZEISS GeminiSEM 500 model devices. XRD analyses were performed by means of Philips PANalytical X’pert Pro and Panalytical Empyrean. BET/BJH analyses were performed using the AUTOSORB-1C/MS.

## 3 Results and Discussion

### 3.1 Synthesized Samples by Using Heated Magnetic Stirrer

A first series of SCS experiments were conducted using different glycine-to-oxidizer ratios ranging from 0.5 to 4 times the stoichiometric requirement. In all cases, the nitrate precursors and glycine were dissolved in water, and the reactions were initiated on a magnetic stirrer preheated to 250 °C. As heating progressed, water gradually evaporated, leading to an increasingly viscous solution, after which combustion was triggered. At a fuel ratio of 0.5×, ignition occurred at approximately the 20th minute, yielding 1.5 g of (CoCrFeMnNi)_3_O_4_. When the stoichiometric ratio was set to 1.0×, combustion began earlier, at about the 10th minute, and produced 1.1 g of product. At a ratio of 1.5× the reaction was initiated after 15 min, yielding 1.32 g, while at a ratio of 2.0×, ignition occurred again around the 10th minute but with visible gas evolution and a comparatively longer reaction duration, resulting in 1.5 g of product. Finally, when the fuel ratio was increased to four times stoichiometry, significant water evaporation occurred, yet no combustion was observed for nearly 45 min, likely due to the excessive amount of glycine, its higher solubility, and incomplete dissolution. The initial and final stages of the reactions were given in Figure 4.

**Figure 4:**
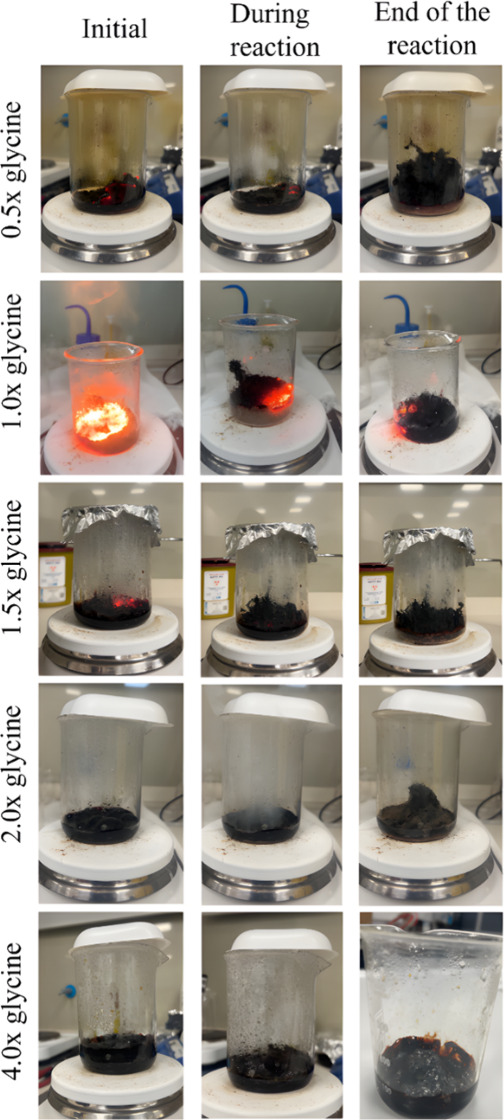
Combustion reactions in SCS experiments with glycine stoichiometry ratios ranging from 0.5× to 4.0×.

Following the SCS experiments conducted with glycine, additional experiments were performed using urea as the fuel at stoichiometric ratios of 0.5×, 1.0×, 1.5×, 2.0×, and 4.0×. In these experiments, the same procedure was used as in the SCS experiments performed with glycine.

At low ratios (0.5× and 1.0×), no combustion was observed; instead, only gas evolution or continuous boiling occurred, and no solid product was obtained. Similarly, when the fuel ratio was 2.0× or increased 4.0×, combustion did not take place, and the solutions merely boiled and solidified without yielding measurable products. In contrast, at a ratio of 1.5×, the combustion reaction was successfully initiated after approximately 20 min, resulting in 1.52 g of product. The initial and final stages of these reactions were shown in Figure 5.

**Figure 5:**
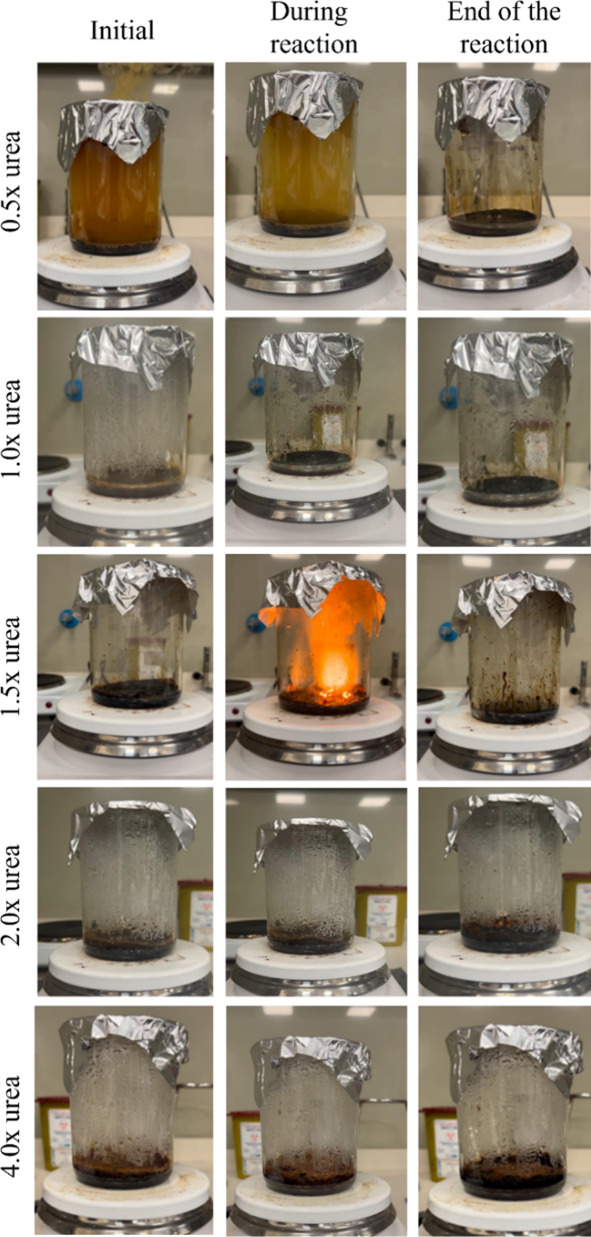
Stages of SCS reactions with urea fuel ratios ranging from 0.5× to 4.0×.

In addition to the effects of glycine and urea on SCS, citric acid has also been studied. At 0.5× times the stoichiometric ratio, no combustion reaction occurred; however, significant gas evolution was observed, and 1.76 g of powder product was obtained after the gas release was completed. At the stoichiometric ratio (1.0×), both combustion and gas evolution took place, yielding 1.34 g of product. When the fuel ratio was increased to 1.5×, only gas evolution was observed, resulting in 1.89 g of product. In contrast, at higher ratios of 2.0× and 4.0×, no combustion occurred and no products were obtained. The stages of these reactions were given in Figure 6. The quantities of products generated following SCS, performed on a heated magnetic stirrer using various fuel types, are summarized in Table 4.

**Figure 6:**
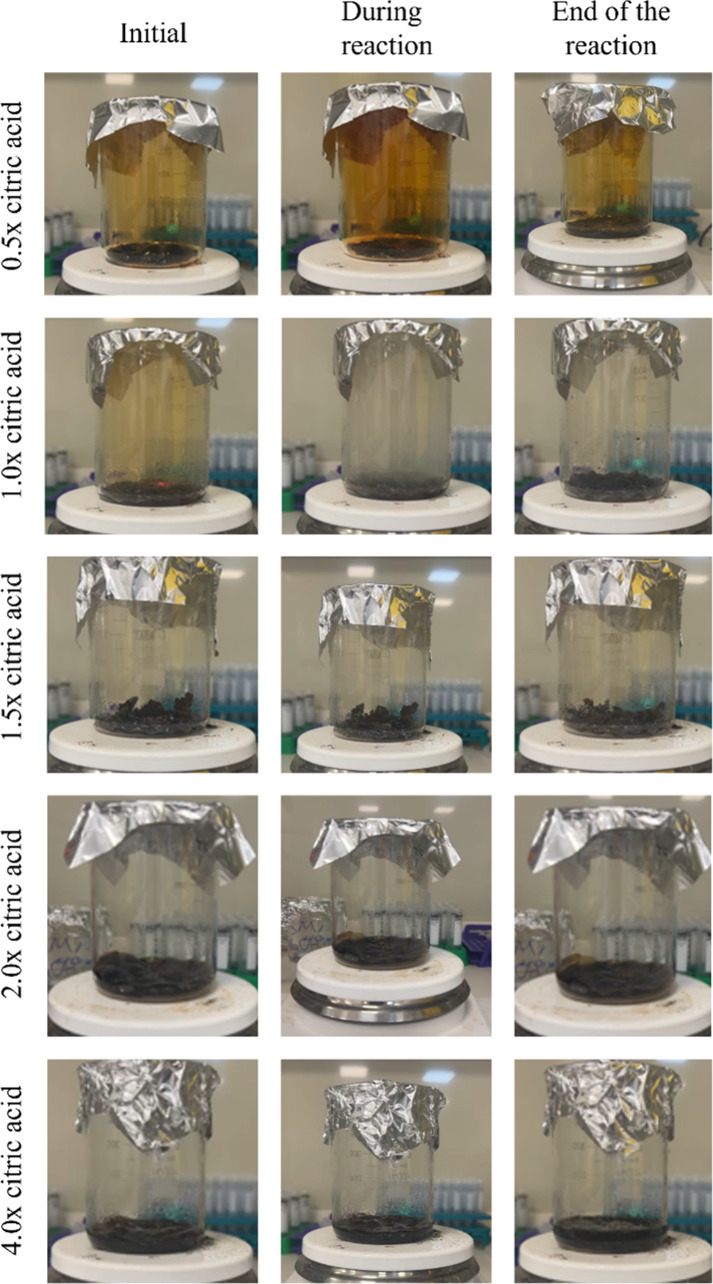
Stages of SCS reactions with citric acid fuel ratios ranging from 0.5× to 4.0×.

**Table 4:** Product Amounts Obtained after SCS in the Heated Magnetic Stirrer

	amount of product (g)				
fuel type	0.5× fuel stoichiometry	1.0× fuel stoichiometry	1.5× fuel stoichiometry	2.0× fuel stoichiometry	4.0× fuel stoichiometry
glycine	1.50	1.10	1.32	1.50	—
urea	—	—	1.52	—	—
citric acid	1.76	1.34	1.89	—	—

The SEM micrographs in Figure 7 reveal that both the type of fuel and the fuel-to-oxidizer ratio exert a significant influence on the microstructure of (CoCrFeMnNi)_3_O_4_ synthesized by SCS. At substoichiometric glycine content (Figure 7a), the combustion was incomplete, resulting in a relatively dense structure with limited porosity. With stoichiometric glycine (Figure 7b), a more homogeneous combustion process generated uniformly distributed pores, leading to a sponge-like morphology with enhanced surface area. Excess glycine (Figure 7c,d) induced vigorous gas release and uncontrolled flame propagation, which produced highly porous yet structurally fragile networks. Elevated fuel-to-oxidizer ratios enhance gas release during combustion, leading to increased porosity and surface area, but the resulting rapid quenching can limit crystallinity.^34^ The porous structure formed as a result of the SCS reaction is due to the release of gaseous products during the combustion reaction.^33^ In contrast, urea at 1.5× ratio (Figure 7e) yielded a more compact and homogeneously porous microstructure, indicating a relatively moderate combustion process compared to glycine. Citric acid–based systems exhibited distinct morphologies: at 0.5×, a dense structure with low porosity was formed (Figure 7f), while at 1.0×, a layered and fibrous morphology appeared (Figure 7g), attributed to the gelation tendency of citric acid during combustion. Increasing citric acid to 1.5× (Figure 7h) resulted in irregular pore structures with large cavities due to nonuniform gas evolution.

**Figure 7:**
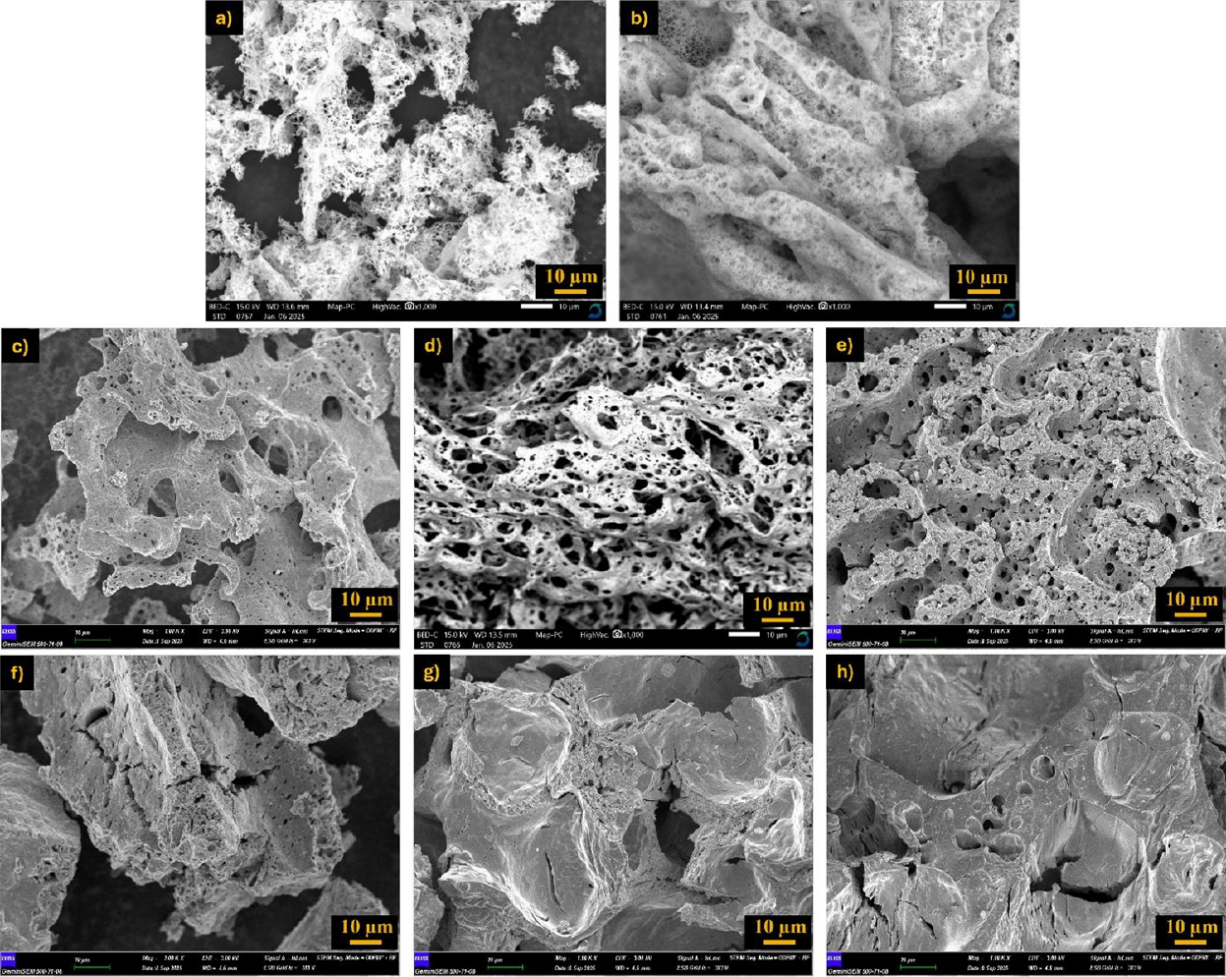
SEM images of products from SCS with different fuels and fuel ratios, synthesized in a magnetic stirrer and taken at 1000× magnification. (a) 0.5× glycine, (b) 1.0× glycine, (c) 1.5× glycine, (d) 2× glycine, (e) 1.5× urea, (f) 0.5× citric acid, (g) 1× citric acid, and (h) 1.5× citric acid.

Table 5 shows the elemental distribution of products obtained with different fuel ratios, based on atomic percentages. In the 0.5×, 1.0×, and 2.0× glycine samples, Cr, Mn, Fe, Co, and Ni were observed to be in the range of approximately 13–16 at. %, and oxygen was at ∼19–24 at. %, indicating that multiple cations were incorporated into the oxide structure in a relatively balanced manner when the fuel ratio was appropriate. In contrast, the 1.5× glycine sample showed a significant oxygen enrichment (52.04 at. %) and a decrease in all metal cations to ∼9–10 at. %, indicating a cation depleted structure consistent with excessive outgassing and porous morphologies. In the 1.5× urea system, the oxygen content was similarly high (56.58 at. %), and the low level of Ni, in particular, indicated a deviation from the ideal co-cation ratio. Samples containing citric acid are the most sensitive to the fuel ratio: at 0.5× citric acid, oxygen predominates (70.84%), and all metal cations are only at ∼5–7 at. %; at 1.0× citric acid, oxygen decreases (35.48 at. %), while Cr and Mn increase relatively, resulting in a more homogeneous distribution; at 1.5×citric acid, the latter again exhibits an oxygen rich (53.33%) and metal-poor structure. In general, the stoichiometric conditions of glycine and citric acid correspond to a more balanced multication oxide structure, while deviations from stoichiometry result in oxygen excess and cation imbalance.

**Table 5:** EDS Data of the Product Obtained with Different Fuel Ratios (*Jeol JCM7000; Others ZEISS GeminiSEM 500)

	amount of content (atomic, %)									
fuel	C	N	O	Al	Cr	Mn	Fe	Co	Ni	Total
0.5× Gly*	7.68	—	21.57	0.36	13.24	13.65	13.96	14.75	14.79	100
1.0× Gly*	4.78	2.14	18.79	0.31	14.56	12.92	15.87	15.40	15.23	100
1.5× Gly	—	—	52.04	—	10.61	9.17	10.18	9.09	8.91	100
2.0× Gly*	5.44	—	23.59	0.24	13.89	13.90	14.72	14.46	13.76	100
1.5 Urea	—	—	56.58	—	10.36	8.80	9.01	8.30	6.95	100
0.5× CA	—	—	70.84	—	6.71	6.05	6.13	5.19	5.08	100
1.0× CA	—	—	35.48	—	15.51	16.09	11.88	11.15	9.89	100
1.5× CA	—	—	53.33	—	10.74	10.52	9.67	8.24	7.50	100

Glycine is a commonly used fuel in solution combustion synthesis due to its high coordination ability with nitrates, low cost, and strong exothermic reaction during combustion. Unlike urea, which contains two amino groups, glycine’s molecular structure consists of an amino group and a carboxylic group located at opposite ends. Owing to the presence of carbon bonds in its structure, glycine is more likely to introduce carbon contamination into the final product compared to urea. Furthermore, under fuel-rich conditions, compared to stoichiometric conditions, the excess fuel increases the likelihood of impurities, such as carbon residues and carbonates, being present in the final product. The carbon structures observed in the EDS results can be attributed to these reasons.^33,35^ In addition, during the SEM-EDS analysis, the carbon material of the tape, the aluminum stub used and the oxygen originating from the environment affect the EDS results.

Overall, the most favorable composition, in terms of low carbon residue and homogeneous cation distribution, was obtained with stoichiometric 1.0× glycine condition, which also correlated well with the optimized microstructures observed in SEM.

The relative reaction front propagation rate (reaction kinetics) of SCS experiments carried out with 3 different fuels (glycine, urea, citric acid) and 5 different fuel ratios (0.5×, 1.0×, 1.5×, 2.0×, 4.0×) in the heated magnetic stirrer were calculated (Figure 8) (Table 6).

**Figure 8:**
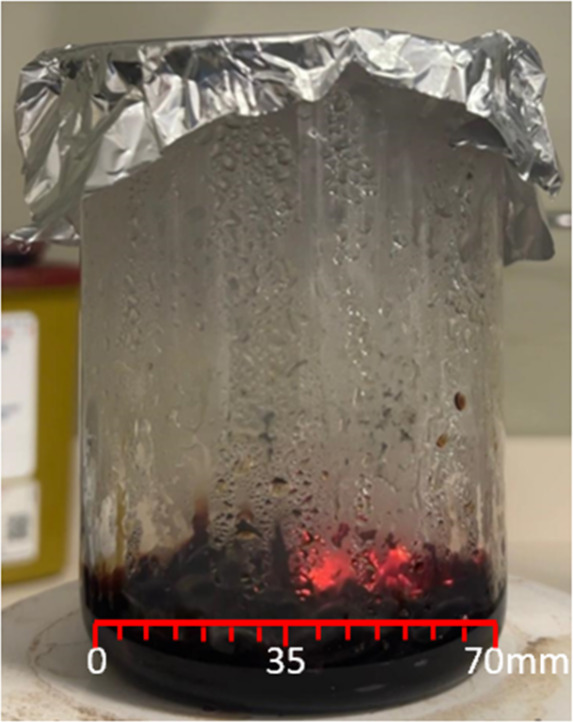
Size of the beaker used in the SCS reactions.

**Table 6:** Relative Reaction Front Propagation Rate (Reaction Kinetics)

type of fuel	fuel ratio	Phi (Φ)	distance traveled (mm)	reaction time (s)	velocity(mm/s)
gycine (C_2_H_5_NO_2_)	0.5	2	70	1	70
	1.0	1	70	2	35
	1.5	0.6	30	2	15
	2.0	0.5	—	—	—
	4.0	0.25	—	—	—
urea (CH_4_N_2_O)	0.5	2	—	—	—
	1.0	1	—	—	—
	1.5	0.6	30	5	6
	2.0	0.5	—	—	—
	4.0	0.25	—	—	—
citric acid (C_6_H_8_O_7_)	0.5	2	—	—	—
	1.0	1	30	4	7.5
	1.5	0.6	—	—	—
	2.0	0.5	—	—	—
	4.0	0.25	—	—	—

In fuel-rich compositions (Φ_e_ < 1), where the fuel content exceeds the stoichiometric ratio, the reaction rate decreases due to an imbalance in the redox system caused by insufficient oxidizer to fully combust the fuel. This imbalance not only slows down the reaction kinetics but also reduces the heat released during combustion, which is critical for sustaining the reaction. Consequently, external heating becomes necessary to provide the additional energy required for the completion of the reaction.^36–38^

In the SCS experiments, products obtained after a complete combustion reaction exhibited magnetic behavior, whereas those formed without combustion showed no magnetism. This distinction demonstrates that fuel type and stoichiometric balance strongly affect both the combustion process and the nature of the final product. Each fuel displayed different combustion characteristics: glycine and citric acid showed high reactivity and energy release, while the performance of urea depended strongly on achieving precise stoichiometric ratios. These results underline the necessity of proper fuel selection and accurate stoichiometry to ensure efficient and reliable SCS synthesis.

XRD analysis was applied to the obtained samples and the results were given in Figure 9. Glycine provided high exothermic energy during the reaction and formed intense and sharp XRD peaks and showed the highest performance in obtaining regular crystal phases. Especially 1.0× glycine ratio provided optimum conditions in terms of regularity and purity of crystal phases. The 0.5× glycine sample synthesized with a low fuel ratio and the 2.0× glycine sample synthesized with a high fuel ratio showed similar XRD patterns. In synthesis without sufficient amount of fuel, combustion may not be complete and therefore low crystallinity may result. In synthesis with excess fuel, the formation of impurities after combustion may occur, which may negatively affect the crystal structure. Urea, although it showed a few peaks, formed amorphous structures. Citric acid, on the other hand, formed more amorphous structures due to its low exothermic reaction capacity and showed limited performance in terms of crystal phase regularity. XRD analysis shows that fuel type and ratio are determinant on the properties of high entropy oxides synthesized by SCS.

**Figure 9:**
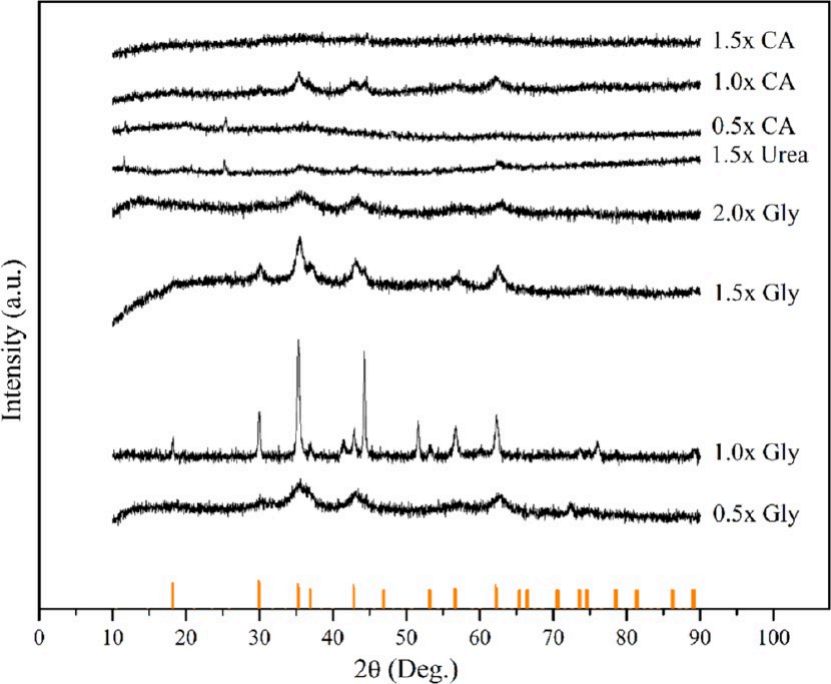
XRD patterns of different SCS products.

In addition, when the XRD pattern of the sample synthesized with a glycine stoichiometric ratio of 1.0× was examined, peak intensities were observed at the 30°–40°, 42°–45° and 52°–65° positions, which correspond well to the spinel structure with space group $Fd\overset{-}{3}m$ , in agreement with the Fe_3_O_4_ reference peaks.

### 3.2 Synthesized Samples by Using the Heating Mantle

After conducting SCS experiments with glycine, urea, and citric acid at various fuel stoichiometry ratios in a heated magnetic stirrer with heating, selected fuel ratios were further investigated using a heating mantle to evaluate the impact of different heating methods on product formation. As shown in Figure 10 and Table 7, the amount of product obtained varied significantly depending on the fuel type and stoichiometry. For glycine, with the highest amount (3.34 g) obtained at a 4.0× fuel ratio. Urea demonstrated optimal combustion at a 1.5× ratio, producing 1.86 g of product, while no combustion was observed at higher ratios. Similarly, citric acid yielded the highest amount of product (2.31 g) at a 1.0× ratio, with decreasing yields observed at 0.5× and 1.5× ratios. The photographs for the initial and medium stages of the reactions were not taken due to closed system of the heating mantle.

**Figure 10:**
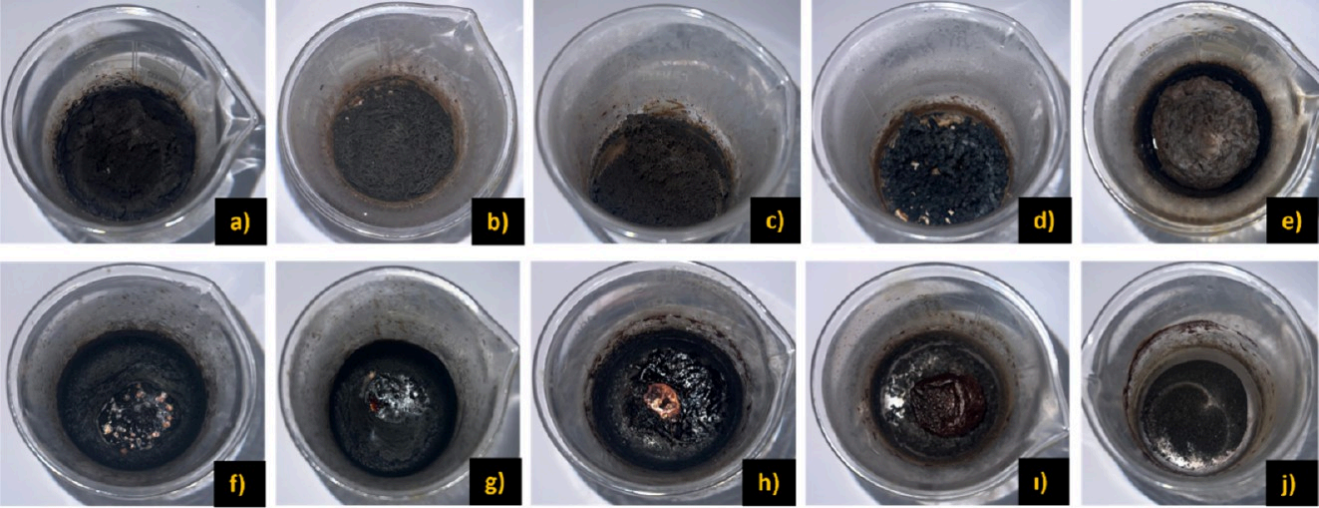
Samples obtained from SCS experiments carried out in the heating mantle. (a) 0.5× glycine, (b) 1.0× glycine, (c) 1.5× glycine, (d) 2.0× glycine, (e) 4.0× glycine, (f) 1.0× urea, (g) 1.5× urea, (h) 0.5× citric acid, (i) 1.0× citric acid, and (j) 1.5× citric acid.

**Table 7:** Product Amounts Obtained after SCS in the Heating Mantle

	amount of product (g)				
fuel type	0.5× fuel stoichiometry	1.0× fuel stoichiometry	1.5× fuel stoichiometry	2.0× fuel stoichiometry	4.0× fuel stoichiometry
glycine	1.51	1.28	1.20	1.33	3.34
urea	—	—	1.86	—	—
citric acid	2.18	2.31	1.81	—	—

When the XRD patterns of the products obtained after SCS experiments with a heating mantle were examined, it was observed that the fuel type and ratio used had significant effects on crystal structure formation (Figure 11). Sharper and more distinct spinel phase peaks were observed, particularly in samples prepared with 1.0× and 1.5× glycine ratios, at positions similar to the Fe_3_O_4_ reference pattern. In samples containing citric acid, XRD peaks were broader and lower in intensity at all fuel ratios, indicating that the combustion behavior of citric acid resulted in high amorphousness and limited crystal growth. Increasing the glycine ratio resulted in increased peak intensities, while in samples using 4.0× glycine and 1.5× urea, the peaks became less pronounced, and the amorphous character became more dominant. These results indicate that a controlled fuel amount (especially 1.0×–1.5× glycine) provides optimal conditions for spinel phase formation, and that excess fuel can negatively affect phase purity.

**Figure 11:**
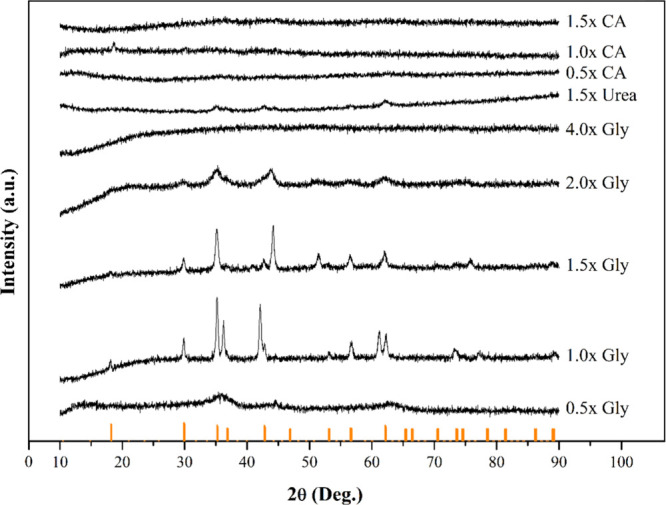
Comparison of XRD patterns of products obtained from SCS experiments. Bars at the bottom represent Fe_3_O_4_ peaks.

### 3.3 Characterization of (CoCrFeMnNi)_3_O_4_ Phases Synthesized by SCS after Calcination

Following the SCS experiments, the calcination step was performed only for fuel compositions that exhibited a self-sustaining combustion reaction. In addition to the macroscopic combustion behavior, XRD and SEM analyses were evaluated to assess phase formation, morphological homogeneity, and the presence of unreacted precursor residues. Based on these combined criteria, the most suitable composition from each fuel type was selected for calcination: 1.0× glycine and 1.5× glycine for glycine-based mixtures, 1.5× urea for urea-based mixtures, and 1.0× citric acid for citric-acid–based mixtures.

Prior to the calcination experiments, TGA was performed on the synthesized (CoCrFeMnNi)_3_O_4_ powders obtained with different fuel types and ratios. Based on the TGA results, calcination temperatures of 800 °C, 900 °C, and 1000 °C, and a time of 1 h were selected to ensure complete removal of volatile components while preserving the oxide structure (Figure 12).

**Figure 12:**
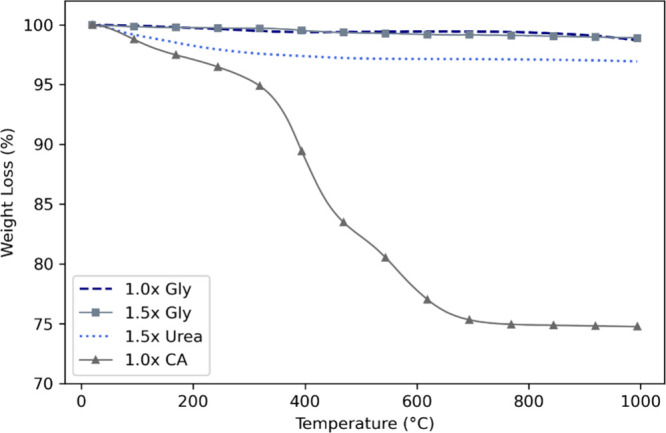
TGA curves of 4 different samples in nitrogen atmosphere.

A comparison of the TGA results (Figure 12) and the amount of product remaining after calcination (Figure 13) reveals consistent trends in both data sets, particularly for the glycine and urea fuelled samples. In TGA analysis, samples prepared with 1.0× glycine, 1.5× glycine, and 1.5× urea showed minimal weight loss (approximately less than 3%) over the entire heating range up to 1000 °C. Correspondingly, in calcination experiments, these samples showed high residual product amounts, ranging from approximately 98% to approximately 111% (Figure 13). This supports the TGA observation that no significant decomposition or mass loss occurred during postsynthesis calcination. During calcination, especially at higher temperatures (900–1000 °C), it is possible for metal oxides to reach higher oxidation states by absorbing oxygen from the environment into the material structure. For example, spinel structures such as (CoCrFeMnNi)_3_O_4_ can bind additional oxygen.

**Figure 13:**
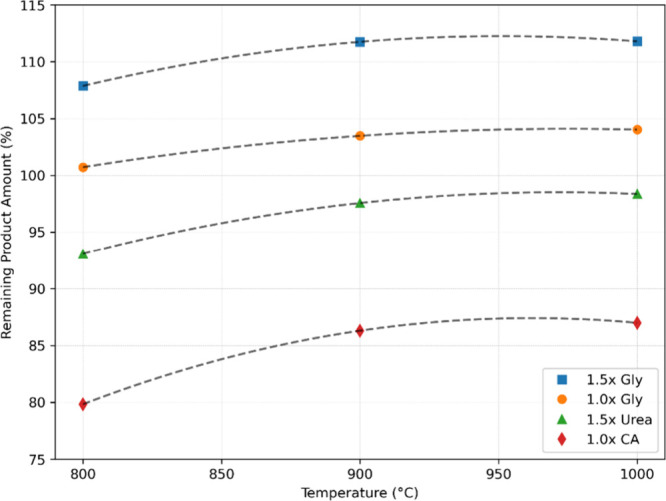
Remaining product amount after calcination.

In contrast, TGA results for the sample synthesized with 1.0× citric acid reveal a significant weight loss (∼25%) between 200 and 700 °C. This suggests the presence of organic matter remaining from the synthesis stage. The calcination results are consistent with this observation: the amount of product remaining in the citric acid sample was significantly lower (80–87%) compared to the other fuels, which coincides with the significant weight loss seen in the TGA.

Before calcination, samples synthesized with only 1.5× glycine and 2.0× glycine exhibit distinguishable diffraction peaks similar to the spinel structure, but with broader and less intense reflections. Samples synthesized with lower fuel ratios (e.g., 0.5× and 1.0× glycine) or 1.5× urea exhibit mostly amorphous properties, with weak or absent peaks, indicating incomplete crystallization during the SCS stage (Figure 11).

After the calcination of the samples, XRD analyses were carried out. Figure 14 shows the XRD patterns after calcination at 800, 900, and 1000 °C. Both series of results were compared to a standard Fe_3_O_4_ spinel reference. At all temperatures and fuel types, diffraction patterns showed similar peaks to the standard Fe_3_O_4_ reference peaks (marked with bars at the bottom) and the formation of the spinel type (CoCrFeMnNi)_3_O_4_ high entropy oxide phase.

**Figure 14:**
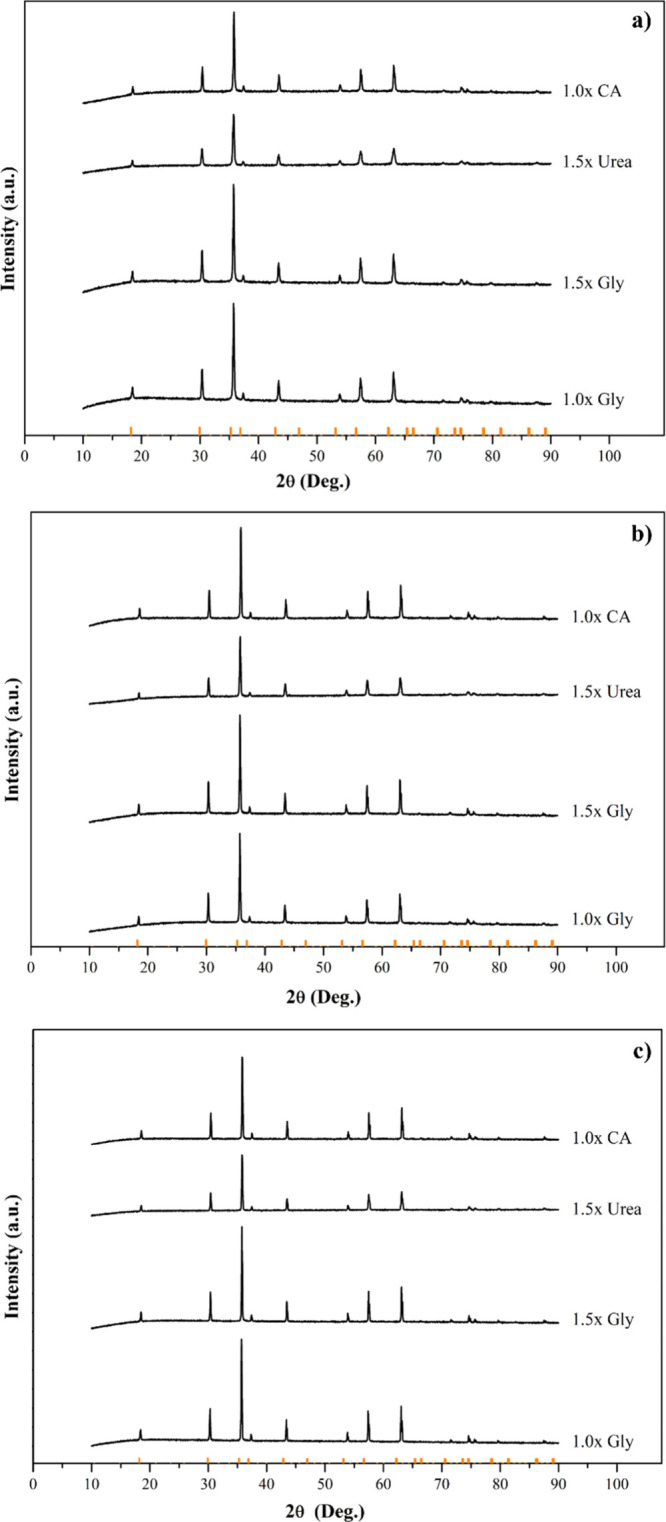
XRD patterns after calcination. (a) @800 °C, (b) @900 °C, and (c) @1000 °C (Bars at the bottom represent Fe_3_O_4_ peaks).

Peak intensities and sharpness increase, particularly at higher calcination temperatures above 900 °C, indicating enhanced crystallization. However, small shifts in peak positions and changes in peak width are observed compared to pure Fe_3_O_4_. These differences are attributed to the multication composition of (CoCrFeMnNi)_3_O_4_, where the substitution of Fe with other transition metals (Co, Cr, Mn, Ni) slightly alters the lattice parameters due to their different ionic radii. Specimens synthesized with 1.0× glycine, in particular, stand out with sharper diffraction peaks at all temperatures. Peak clarity and intensity were found to be lower for other fuel types and ratios.

Considering the weight gain observed during calcination, no additional peaks representing secondary phases such as simple oxides (e.g., Fe_2_O_3_, CoO) or unreacted precursors were detected, confirming the transformation to a high-entropy spinel phase.

In summary, this demonstrates that some spinel formation is possible in glycine-rich samples immediately after SCS, but complete crystallization and phase purity, resembling the Fe_3_O_4_ type spinel structure, are achieved only after calcination. The combination of XRD and calcination results confirms the necessity of thermal treatment for both the formation mechanism and phase stabilization.

Postcalcination SEM analyses revealed that particle sizes increased and the morphology became more compact with increasing temperature (Figures 15–17). Specifically, samples obtained with 1.0× glycine exhibited more homogeneous and smooth-surfaced, and coarse-grained structures at all three temperatures, while 1.5× glycine fuel produced a more porous and irregular morphology. In samples obtained with 1.5× urea and 1.0× citric acid, irregular and fine-grained structures were prominent, particularly at lower temperatures (800 °C), while slightly more pronounced crystalline structures were observed at 1000 °C.

**Figure 15:**
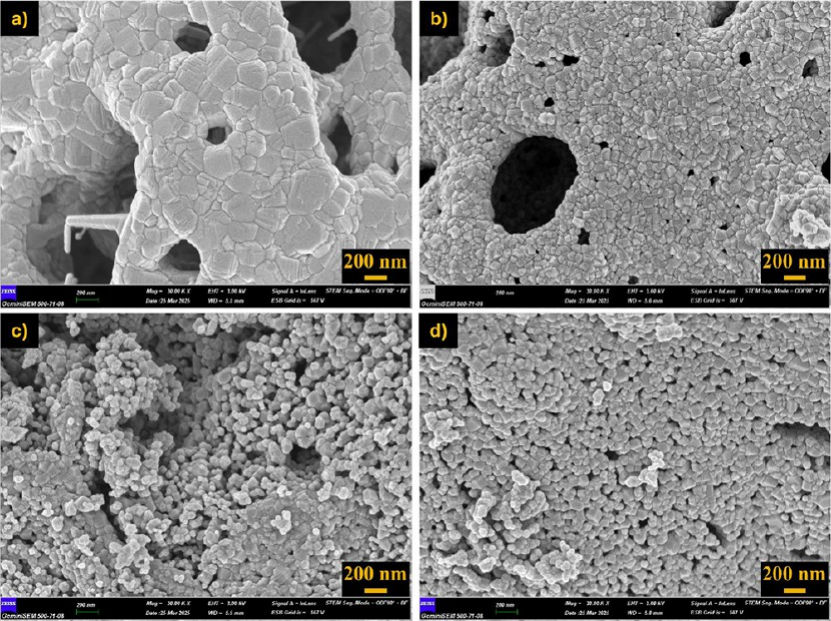
SEM images after calcination at 800 °C for 1 h and x30,000 magnification. (a) 1.0× glycine, (b) 1.5× glycine, (c) 1.5× urea, and (d) 1.0× citric acid.

**16:**
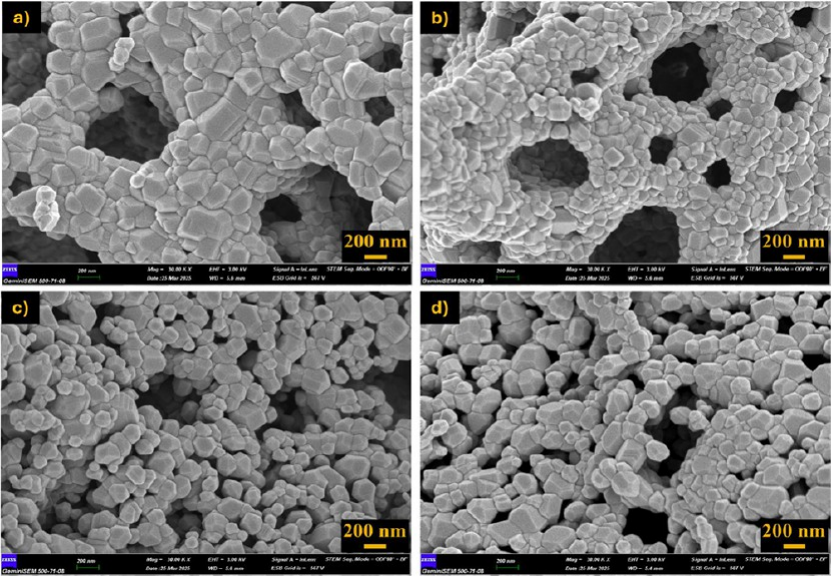
SEM images after calcination at 900 °C for 1 h and ×30,000 magnification. (a) 1.0× glycine, (b) 1.5× glycine, (c) 1.5× urea, and (d) 1.0× citric acid.

**Figure 17:**
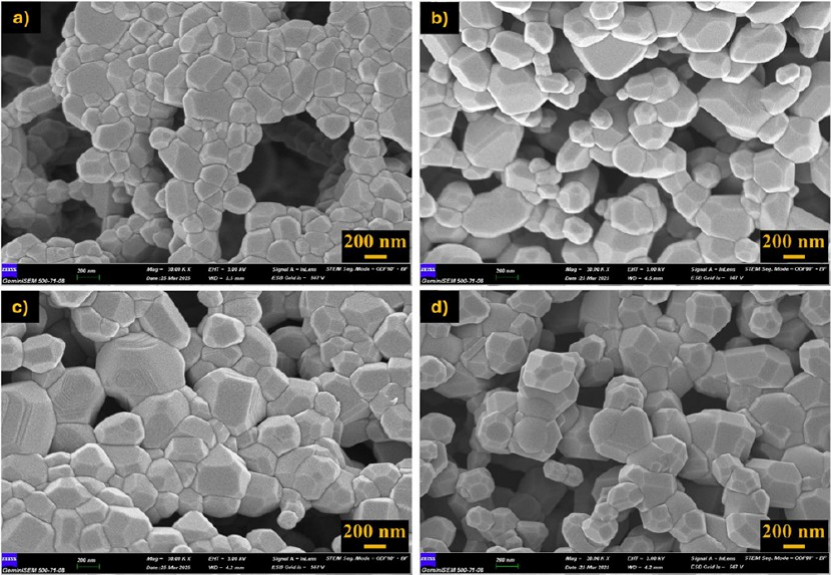
SEM images after calcination at 1000 °C for 1 h and ×30,000 magnification. (a) 1.0× glycine, (b) 1.5× glycine, (c) 1.5× urea, and (d) 1.0× citric acid.

A general evaluation of Table 8 shows that the elemental distribution after calcination is sensitive to both fuel type and temperature. At 800 °C, the 1.0× glycine and 1.5× glycine samples exhibited the closest and most balanced cation distributions in terms of Cr, Mn, Fe, Co, and Ni, while 1.5× glycine exhibited a slightly cation-diluted structure with high oxygen content, and 1.5× urea exhibited low oxygen and a Cr/Mn-rich and Ni-poor surface composition. The increase in oxygen content in the 1.0× glycine sample calcined at 900 °C is consistent with more advanced oxidation on the surface. At 1000 °C, especially under urea and excess fuel conditions, Ni remained significantly lower, and Cr/Mn enrichment continued.

**Table 8:** EDS Data of Samples Calcined at Different Temperatures

		amount of content (atomic, %)					
temperature (°C)	fuel	O	Cr	Mn	Fe	Co	Ni
800	1.0× Gly	42.01	12.47	11.95	11.65	11.29	10.62
	1.5× Gly	47.38	11.54	11.24	10.48	10.01	9.35
	1.5× Urea	33.88	15.86	15.37	13.93	12.84	11.49
	1.0× CA	44.42	12.49	12.08	11.01	10.28	9.73
900	1.0× Gly	49.10	10.71	10.96	10.36	9.57	9.29
	1.5× Gly	30.52	15.74	15.30	13.91	12.85	11.67
	1.5× Urea	32.74	15.85	15.52	12.59	12.35	10.95
	1.0× CA	34.97	15.59	15.19	12.85	11.36	10.04
1000	1.0× Gly	39.45	13.46	13.02	12.21	11.25	10.60
	1.5× Gly	41.76	13.36	12.79	11.56	10.68	9.86
	1.5× Urea	44.59	12.84	10.69	13.97	9.14	8.78
	1.0× CA	38.03	14.47	13.77	12.32	11.29	10.12

Table 9 shows that both fuel chemistry and calcination temperature affect the normalized atomic distributions of the spinel oxide components. At 800 °C, the 1.5× glycine and 1.0× citric acid samples exhibit a more even distribution. However, fuel-induced differences emerge: urea-rich formulations show slight Cr–Mn enrichment, while glycine- and citric acid–based samples display more uniform multication incorporation. Increasing the fuel fraction (e.g., 1.5× glycine or 1.5× urea) tends to marginally shift the oxide equilibrium toward Cr_3_O_4_ and Mn_3_O_4_, suggesting that fuel-rich conditions favor their relative stabilization within the spinel lattice. At 900 °C, the 1.0× glycine sample also shows a notably homogeneous distribution, indicating that near-stoichiometric fuel ratios promote cation uniformity during thermal restructuring. As the calcination temperature increases further to 1000 °C, fuel-derived differences gradually diminish, and the oxide fractions coalesce, reflecting enhanced cation diffusion and a more stable high-entropy spinel framework.

**Table 9:** Normalized EDS Data of Samples Sintered at 3 Different Temperatures (800 °C, 900 °C, 1000 °C Atomic, %)

		amount of content (atomic, %)				
temperature (°C)	fuel	Cr_3_O_4_	Mn_3_O_4_	Fe_3_O_4_	Co_3_O_4_	Ni_3_O_4_
800	1.0× Gly	21.51	20.61	20.09	19.47	18.32
	1.5× Gly	21.93	21.36	19.92	21.46	20.18
	1.5× Urea	22.82	22.12	20.05	16.25	15.28
	1.0× CA	22.47	21.73	19.81	20.31	19.10
900	1.0× Gly	21.05	21.54	20.36	22.19	20.87
	1.5× Gly	22.66	22.02	20.02	16.25	15.29
	1.5× Urea	23.57	23.07	18.72	16.79	15.79
	1.0× CA	23.97	23.36	19.76	17.36	16.33
1000	1.0× Gly	22.23	21.51	20.17	18.65	17.54
	1.5× Gly	22.94	21.96	19.85	19.38	18.23
	1.5× Urea	23.17	19.29	25.21	20.37	19.16
	1.0× CA	23.35	22.22	19.88	18.22	17.14

In summary, when the weight change after calcination, XRD, and SEM-EDS analyses were evaluated together, it was determined that 800 °C 1.0× and 1.5× glycine and 900 °C 1.0× glycine to fuel ratio of (CoCrFeMnNi)_3_O_4_ provided the optimum conditions for high entropy oxides. In samples obtained under these conditions, sharp and intense spinel peaks were observed in XRD patterns, indicating high phase purity, while SEM images revealed a homogeneously distributed, controlled sized crystal morphology. EDS results also showed that samples prepared with 1.0× glycine had values closest to theoretical equimolarity in terms of elemental distribution. These parameters provide the optimal conditions for both maintaining crystal structure integrity and achieving the desired microstructural properties.

Based on findings, BET surface area analysis was further performed on the optimized glycine-derived samples prepared at a fixed fuel stoichiometry of 1.0× and calcined at 800, 900, and 1000 °C. This analysis was conducted to quantitatively evaluate the evolution of surface area, pore size, and pore volume as a function of calcination temperature and to provide numerical support for the microstructural features observed by SEM. Postcalcination SEM observations indicated progressive grain growth and a more compact morphology with increasing temperature. This trend is quantitatively supported by BET/BJH analysis for the 1.0× glycine series (Table 10), where the specific surface area decreased from 18.80 m^2^/g (800 °C) to 13.48 m^2^/g (900 °C) and further to 3.20 m^2^/g (1000 °C). Although grain growth is evident, the concurrent increase in BJH pore diameter from ∼2.52 to ∼5.80 nm reflects pore coarsening due to the coalescence and closure of smaller pores rather than an increase in grain size. In parallel, the mesopore volume remained nearly constant between 800 and 900 °C (∼0.047 cm^3^/g) but decreased markedly at 1000 °C (∼0.021 cm^3^/g), indicating advanced densification accompanied by a significant reduction in accessible porosity, fully consistent with SEM observations.

**Table 10:** Textural Properties of Glycine-Derived (CoCrFeMnNi)_3_O_4_ Calcined at Different Temperatures

fuel	calcination (°C)	S_BET_ (m^2^/g)	BJH pore diameter (nm)	BJH pore volume (cm^3^/g)
1.0× glycine	800	18.80	2.52	0.04741
	900	13.48	2.81	0.04721
	1000	3.20	5.80	0.02111

Additionally, in order to evaluate possible color changes that may occur in the samples after the calcination process, visual records of the samples for each temperature and fuel stoichiometry condition were taken and comparative observations were made (Figure 18).

**Figure 18:**
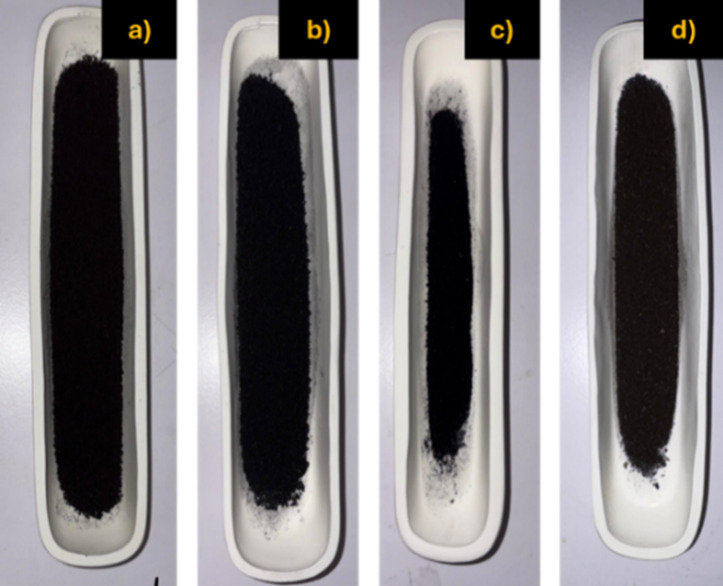
Sample images before calcination. (a) 1.0× glycine, (b) 1.5× glycine, (c) 1.5× urea, and (d) 1.0× citric acid.

As shown in Figure 19, visual inspection of the calcined samples revealed no visible differences depending on the fuel type and calcination temperature.

**Figure 19:**
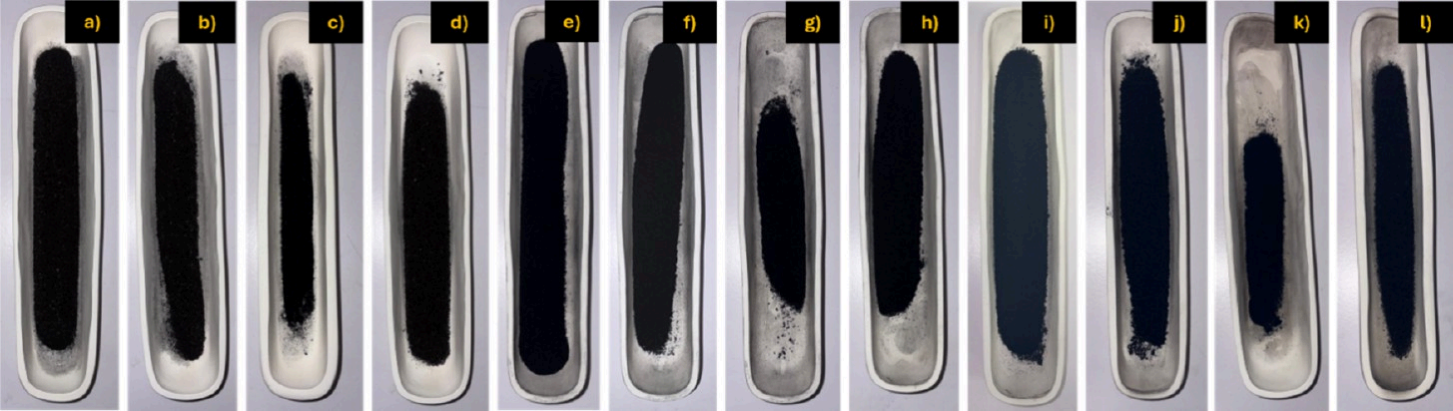
Sample images after calcination for 1 h at three different temperatures. (a) 1× glycine @800 °C, (b) 1.5× glycine @800 °C, (c) 1.5× urea @800 °C, (d) 1.0× citric acid @800 °C, (e) 1× glycine @900 °C, (f) 1.5× glycine @900 °C, (g) 1.5× urea @900 °C, (h) 1.0× citric acid @900 °C, (i) 1.0× glycine @1000 °C, (j) 1.5× glycine @1000 °C, (k) 1.5× urea @1000 °C, (l) 1.0× citric acid @1000 °C.

## 4 Conclusion

This study systematically investigated the synthesis of high-entropy spinel oxide (CoCrFeMnNi)_3_O_4_ through SCS using three different fuels (glycine, urea and citric acid) at multiple fuel-to-oxidizer ratios, followed by calcination between 800 and 1000 °C. The experimental results clearly demonstrate that the combustion characteristics, phase stability and microstructural features of the products are highly dependent on the type and stoichiometry of the fuel.

Among the investigated fuels, glycine exhibited the most favorable performance, particularly at the stoichiometric ratio, yielding sharp and well-defined diffraction peaks corresponding to the $Fd\overset{-}{3}m$ spinel structure, along with homogeneous elemental distribution close to theoretical equimolarity. Urea was found to promote phase formation only within a narrow compositional window, while citric acid resulted in incomplete combustion, residual carbonaceous phases and reduced phase purity, as corroborated by TGA and SEM-EDS analyses.

Post-SCS thermal treatment was essential for stabilizing the single-phase spinel structure. Calcination at 900 °C provided the optimal balance between crystallinity, phase purity and controlled grain growth, while higher temperatures promoted further densification without significant improvements in homogeneity. BET and BJH analyses of the glycine-derived samples prepared at the 1.0× fuel stoichiometry confirmed the temperature-dependent evolution of surface area and porosity, showing a marked decrease in specific surface area and pore volume with increasing calcination temperature, in line with the overall densification observed by SEM. The absence of secondary oxides after calcination confirmed the successful stabilization of the high-entropy spinel phase.

Overall, the findings establish a direct correlation between fuel chemistry, combustion dynamics and final material characteristics, thereby defining processing window for obtaining phase-pure (CoCrFeMnNi)_3_O_4_ at relatively low processing temperatures. This work expands the understanding of fuel-dependent SCS routes for high-entropy oxides and provides an experimental framework for tailoring their microstructural and functional properties for future applications in energy storage, catalysis and magnetic technologies.
